# FEVR combined with macular heterotopia in children presenting as pseudo-exotropia: a case report and literature review

**DOI:** 10.3389/fmed.2024.1409074

**Published:** 2024-08-26

**Authors:** Yujie Zhang, Shuimiao Chen, Yanghui Xiu

**Affiliations:** ^1^Xiamen Eye Center and Eye Institute of Xiamen University, Xiamen, China; ^2^Xiamen Clinical Research Center for Eye Diseases, Xiamen, Fujian, China; ^3^Xiamen Key Laboratory of Ophthalmology, Xiamen, Fujian, China; ^4^Fujian Key Laboratory of Corneal and Ocular Surface Diseases, Xiamen, Fujian, China; ^5^Xiamen Key Laboratory of Corneal and Ocular Surface Diseases, Xiamen, Fujian, China; ^6^Translational Medicine Institute of Xiamen Eye Center of Xiamen University, Xiamen, Fujian, China

**Keywords:** familial exudative vitreoretinopathy, macular heterotopia, pseudo-strabismus, binocular vision, macular perfusion density evaluation

## Abstract

Familial exudative retinopathy (FEVR) is a hereditary disease involving abnormal retinal vascular development in which macular heterotopia (MH) caused by mechanical-like pulling of the vitreous may lead to pseudo-strabismus. We describe the case of a 12-year-old male patient from China who presented to our hospital with a request for surgical correction of exotropia. Examination revealed that the strabismic appearance was due to MH, and dilated pupil examination of the peripheral fundus revealed that the blood vessels of the left eye and the macula were displaced toward the temporal retina by pulling, and further FFA examination was performed to diagnose FEVR. With good binocular vision and stereoscopic distance vision, corrective surgery for strabismus in this patient would have resulted in a hard-to-resolve diplopia. Therefore, it is important to identify FEVR combined with MH in clinical practice to avoid wrong diagnostic and treatment options.

## Introduction

Familial exudative vitreoretinopathy (FEVR) is a genetic disease characterized by abnormal development of retinal vessels, mainly affecting the generation of retinal vessels and leading to poor differentiation of peripheral retinal vessels, with diverse clinical features (1). Typical clinical features include avascular areas around the retina, arteriovenous shunt formation, macular and vascular traction, vitreous retinal adhesion, retinal folds, retinal exudation, and even retinal detachment (RD) (2). If any eye of a full-term or preterm infant has peripheral retinal avascularity and the disease process is inconsistent with retinopathy of prematurity (ROP), it can be diagnosed as FEVR (3). The eyes of patients with FEVR can exhibit a wide range of symptoms, from mild peripheral vessel formation in the stationary phase to complete retinal detachment with irreversible loss of vision in the later stage, and when vitreous traction is induced, it can lead to an abnormal position of the macular, resulting in macular heterotopia (4).

The macular fovea is located temporally inferior to the center of the optic disc in most eyes (5). Macular heterotopia (MH) is the traction of vitreoretinal on fibrovascular and retinal tissue resulting in macular displacement to a different retinal position. The etiology of MH is diverse, including choroidal retinitis (6), vitreous vascular remnants (7), FEVR (8), ROP (9, 10), parasitic infections (11), diabetic retinopathy (12), and Riley-Day syndrome (13). The macula is the center of visual fixation. If the position of the macula changes, it will cause a change in the angle between the visual axis and the ocular axis, leading to “pseudo-strabismus.” Pseudo-strabismus refers to a condition in which the patient’s appearance shows strabismus, but the alternative cover test (ACT) does not show strabismus. For example, infants and young children with wide nasal bridges and epicanthus may have pseudo-esotropia. In addition, an excessively large kappa angle may also lead to pseudo-strabismus.

Angle Kappa refers to the angle between the visual axis (the line connecting the fixation point and the fovea centralis) and the optical axis (the line connecting the cornea center and the posterior pole of the retina). In most people, the fovea centralis is slightly deviated from the optical axis. If the position of the fovea centralis is on the temporal side of the optical axis, it is a positive angle Kappa; if it is on the nasal side of the optical axis, it is a negative angle Kappa, with an average angle Kappa of 4–5° (14). In general, the angle Kappa is essentially the same in both eyes. Therefore, when the macular is significantly deviated from its normal position, leading to asymmetric spacing between the macular of both eyes and the optic disc, the angle Kappa of both eyes may be asymmetric. This may result in a “strabismus” appearance. A positive large angle Kappa may lead to pseudo-exotropia, while a negative large angle Kappa may result in pseudo-esotropia (15–17). Now a case of pseudo-exotropia caused by FEVR complicated with MH is reported.

## Case report

**Figure 1:**
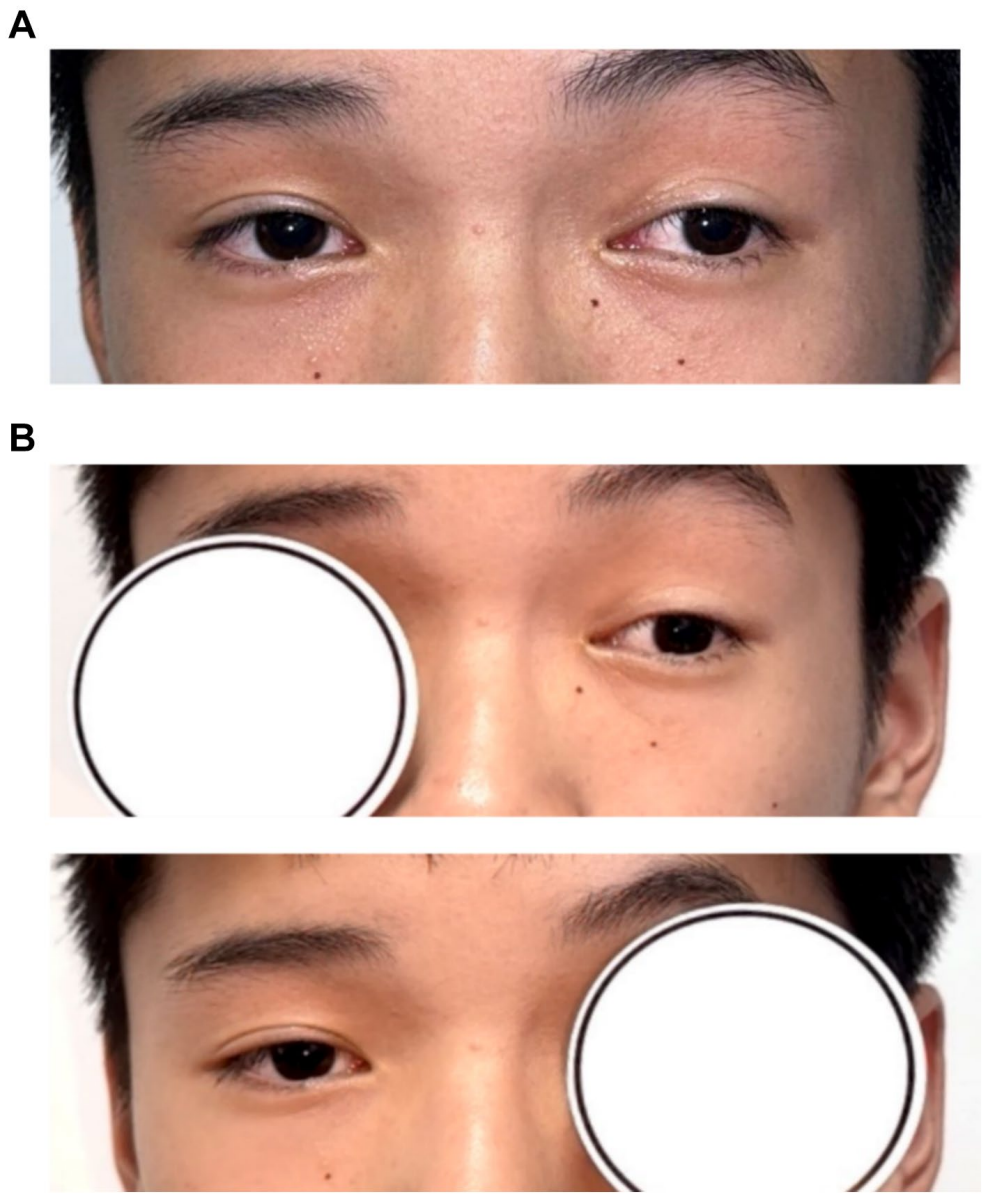
Patient’s eye position. **(A)** Hirschberg method shows −15° in the left eye; **(B)** alternate masking: slight external shadow motion, motion inconsistent with corneal reflection point.

**Figure 2:**
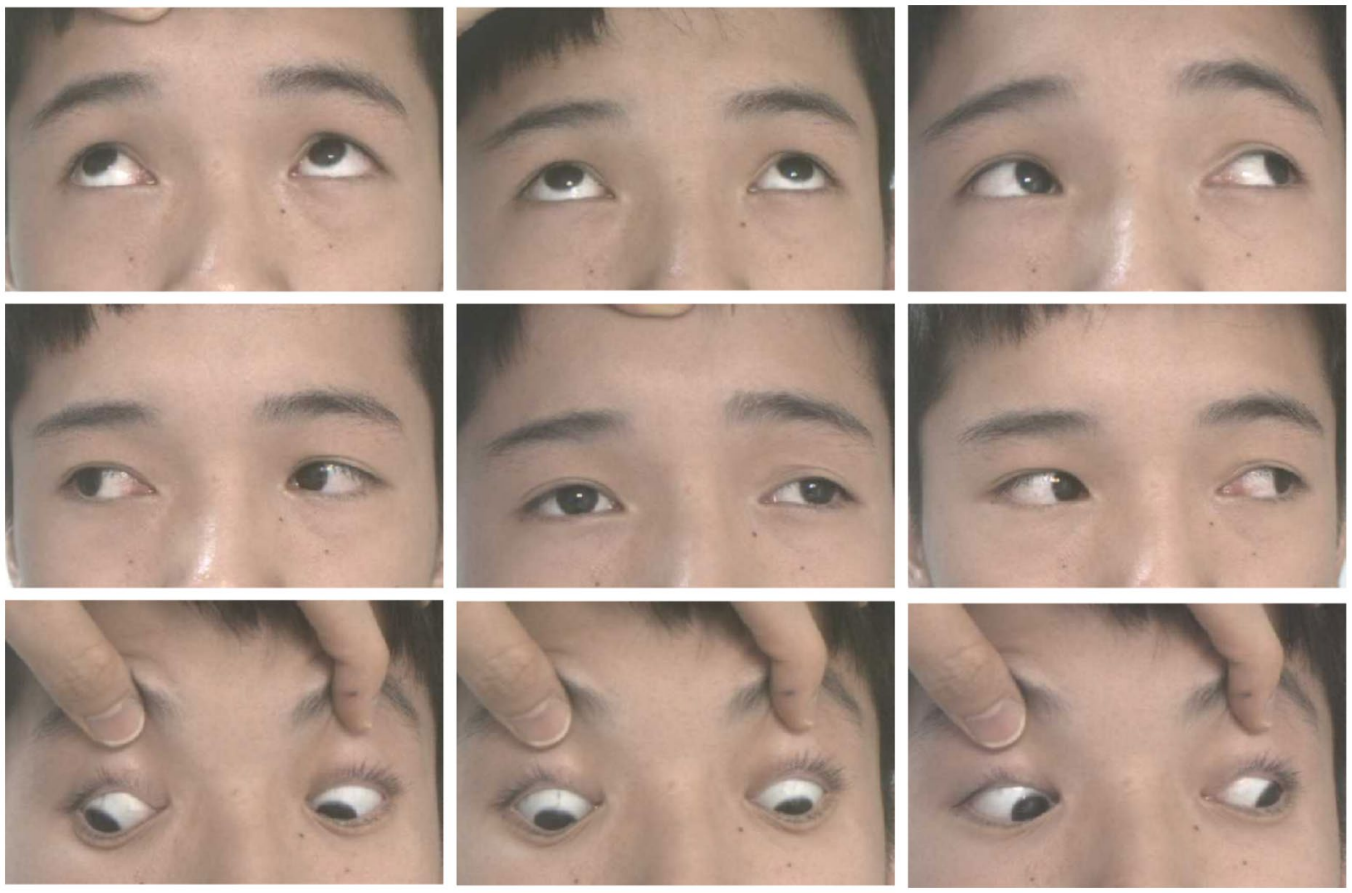
Nine direction eye situation. Normal movement of both eyes in all directions.

**Figure 3:**
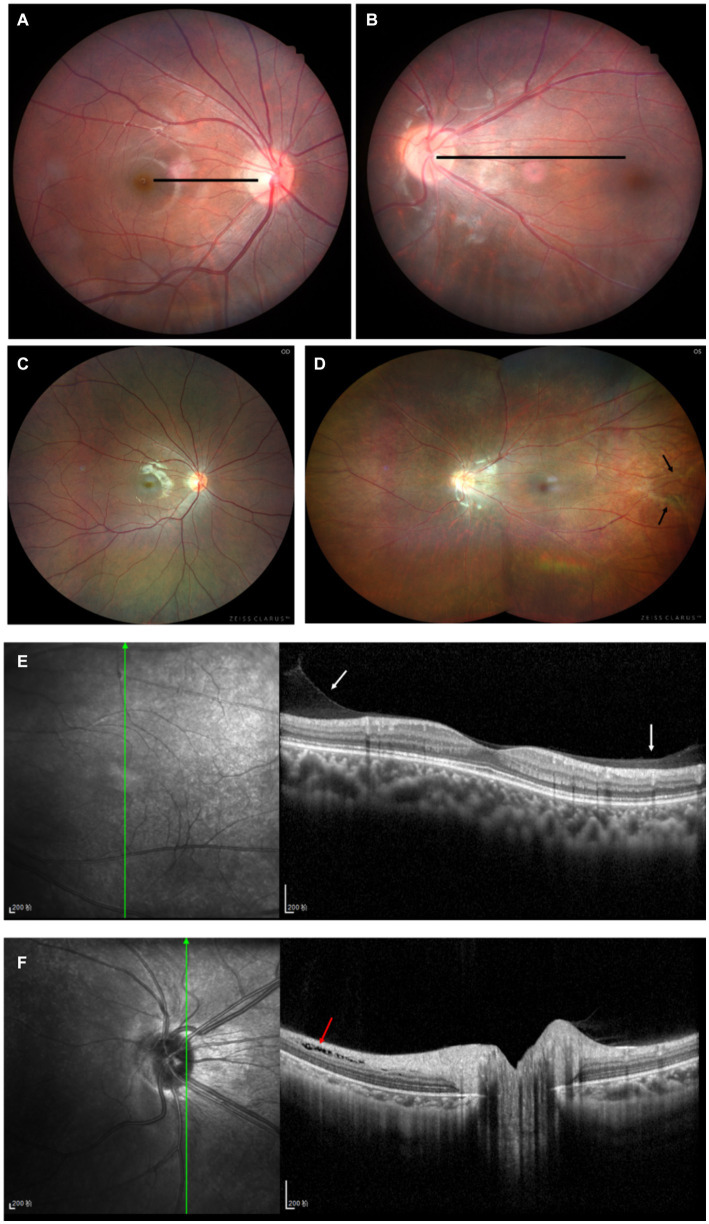
Patient’s fundus condition. **(A,B)** For fundus photography: no obvious abnormality in the right eye **(A)**, the macula in the left eye is shifted to the temporal side, and the macula is 5 PD from the optic disc on the temporal side **(B)**. **(C,D)** For Zeiss wide-angle fundus photography: no obvious abnormality in the right eye’s peripheral retina **(C)**, the vascular arch of the left eye’s retina is straightened to the temporal side, and the black arrows show the pre-fibrofilarial membrane-like hyperplasia of the temporal side of the peripheral retina **(D)**. **(E,F)** For the left eye’s posterior segment. OCT, macular morphology is fair, white arrow shows a little fibrous membranous proliferation, red arrow shows local separation between neuroepithelial layers.

**Figure 4:**
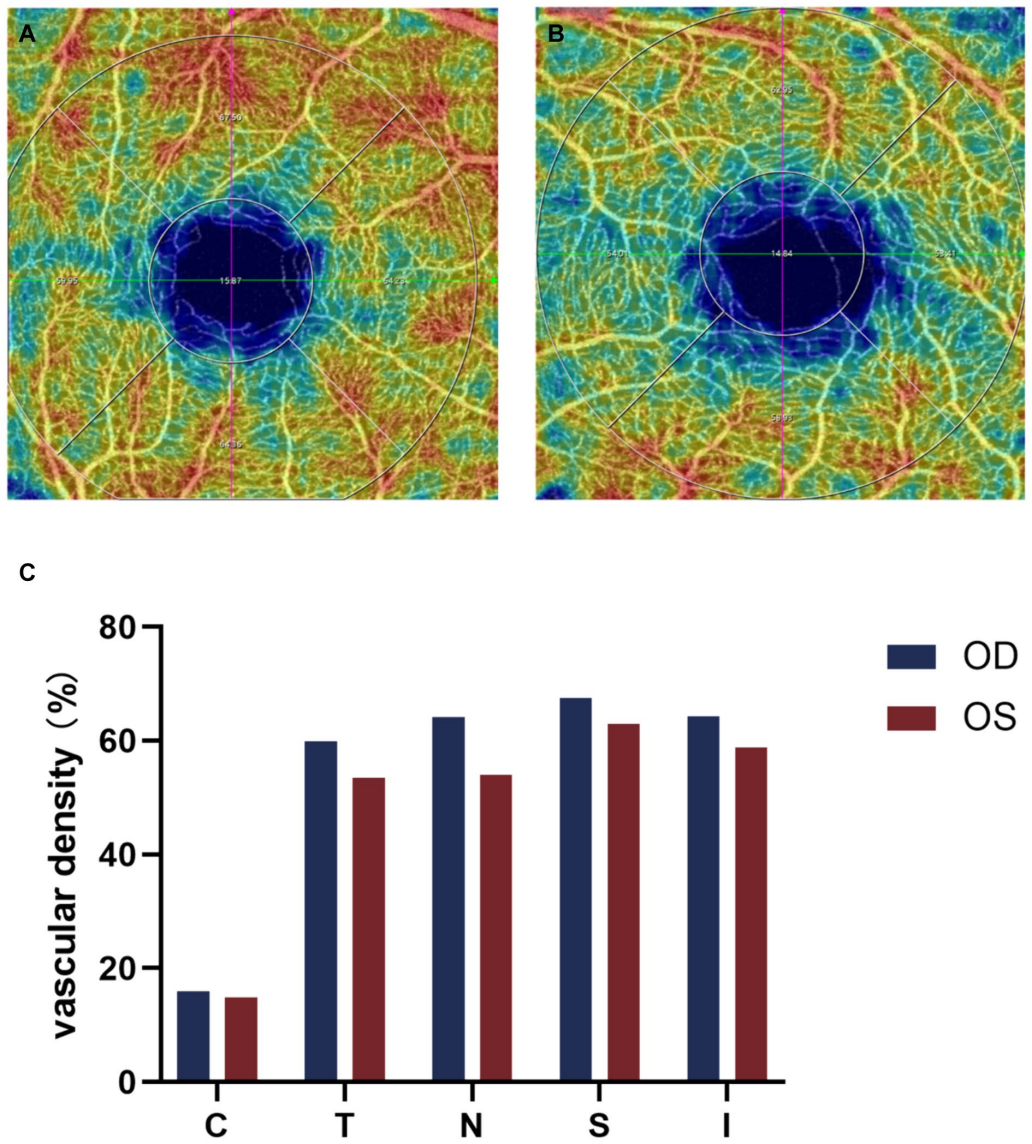
Binocular OCTA macular perfusion density evaluation. **(A,B)** OCTA macular blood flow density imaging, **(C)** Comparison of macular perfusion density in each region of both eyes, the macular perfusion density of the left eye is lower than that of the right eye, and the decrease is more pronounced on the nasal side. C, center; T, temporal; N, nasal; S, superior; I, inferior.

**Figure 5:**
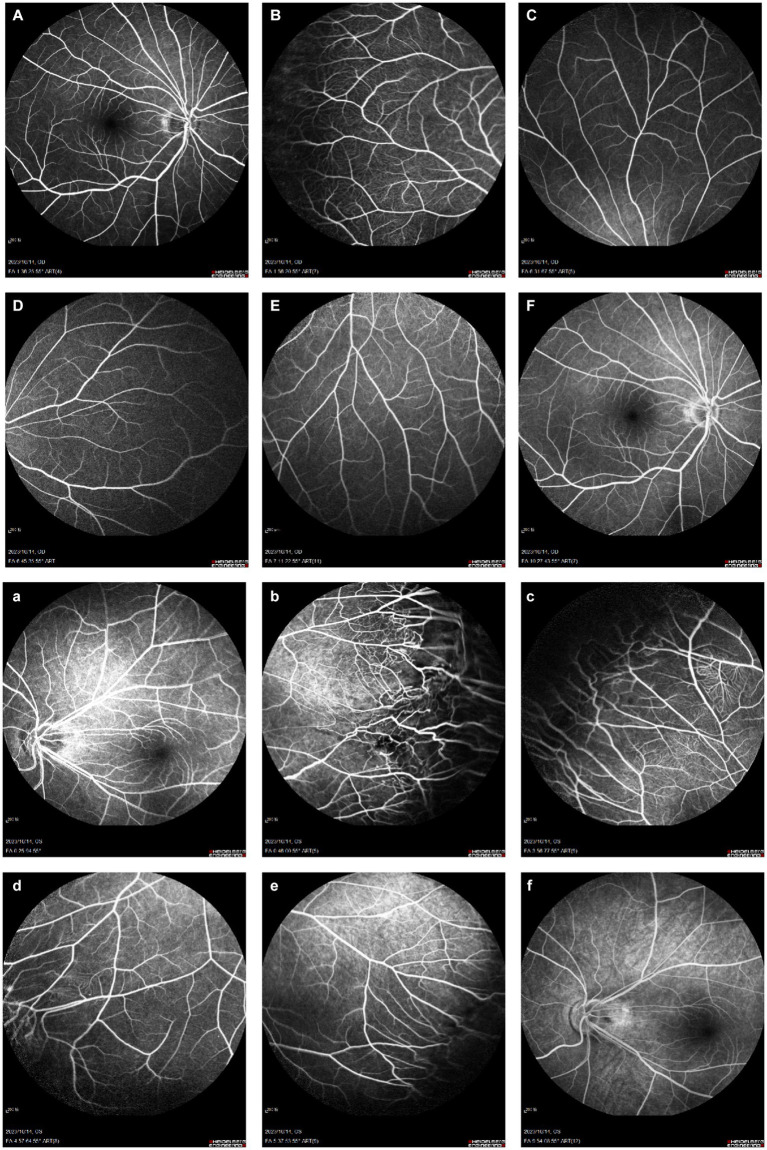
Binocular FFA. **(A–F)** Right eye: early temporal peripheral retinal surface with tortuous vascular alignment; **(a–f)** Left eye: early mid-peripheral retinal vascular alignment is straight, peripheral retinal vascular alignment is tortuous, tube diameter is not uniform, capillary dilation, with the imaging process, the dilated capillaries and part of the vessel wall appear fluorescence leakage, and fluorescence staining in the late stage.

**Figure 6:**
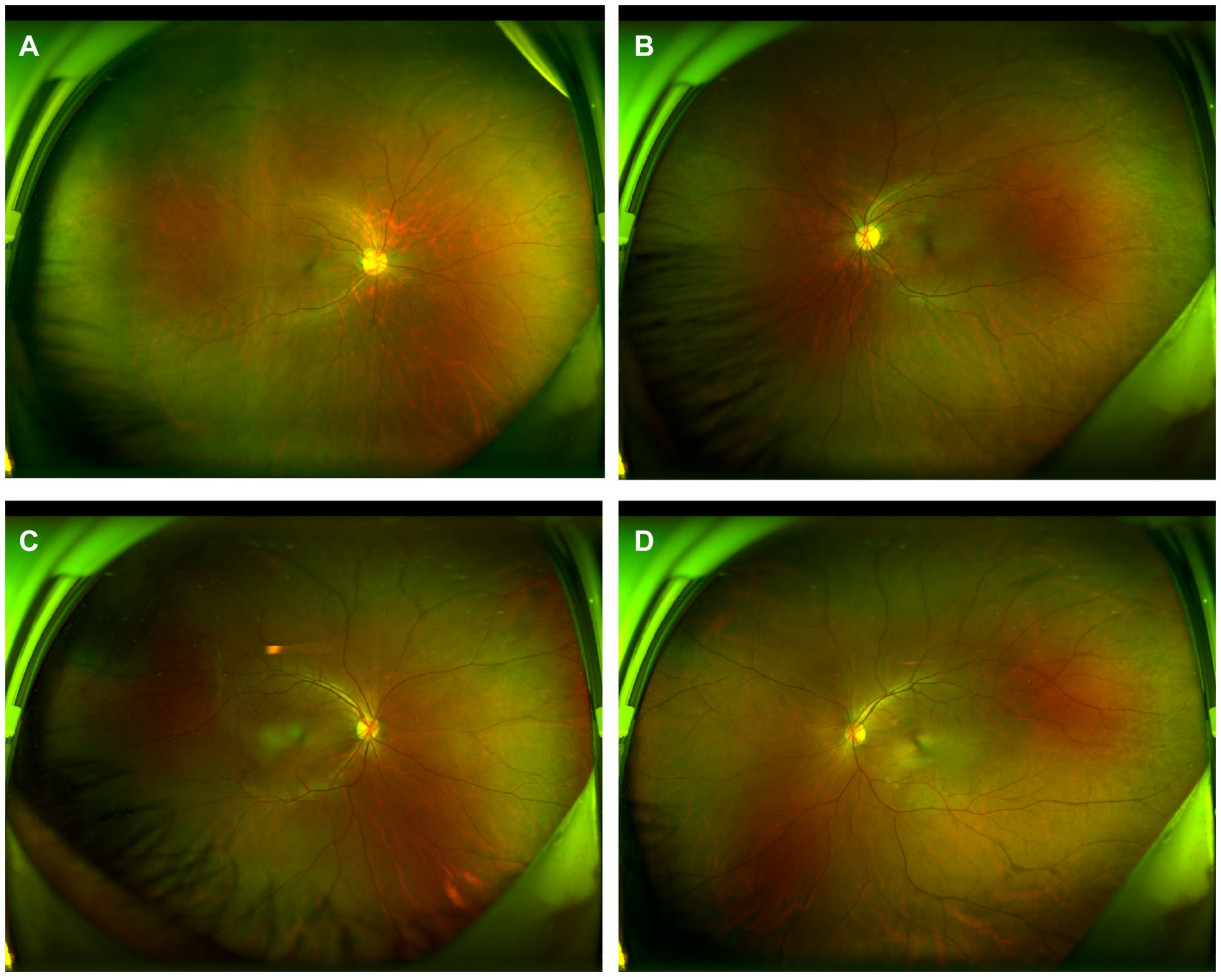
Wide-angle fundus photography of the patient’s parents. Panels **(A,B)** are fundus photographs of the patient’s mother, and Panels **(C,D)** are fundus photographs of the patient’s father, all of which show no significant abnormalities.

A 12-year-old male patient who referred to our pediatric ophthalmology clinic at the Xiamen Eye Center of Xiamen University with chief complaint of “noticed left eye outward deviation persisting for over 7 months.” The initial manifestation of the patient’s left eye deviation was observed during a routine glasses fitting at a local optician 7 months prior. The patient did not report any accompanying symptoms such as diplopia, ocular redness, or pain. The initial diagnosis, recorded as “exotropia,” was made without the administration of any therapeutic intervention, prompting the recommendation for further evaluation and management at a tertiary care facility. The patient’s birth history was unremarkable, with full-term, spontaneous delivery and no history of oxygen supplementation, febrile seizures, or ocular trauma. Additionally, there was no reported history of consanguineous marriage among his parents, and no familial predisposition to strabismus was identified. Upon physical examination, no significant systemic abnormalities were detected. Ophthalmological assessment revealed a distance visual acuity of 20/30 in the right eye and 20/32 in the left eye. Compound tropicamide cycloplegic refraction corrected the visual acuity to 20/20 in the right eye with a refraction of −0.50DS/−0.50 DC × 175, and to 20/30 in the left eye with a refraction of −1.00 DC × 45°. Intraocular pressure measurements were within normal limits, at 15.6 mmHg in the right eye and 16.8 mmHg in the left eye. Alignment assessment using the 33 cm corneal reflection method indicated a left eye deviation of −15° (Figure 1A). Further evaluation with the cover-uncover and alternate cover test revealed a slight external shadow motion that was incongruent with the corneal reflection point (Figure 1B). Binocular prism cover testing at 33 cm necessitated a base-in 20Δ to base-out 20Δ with diplopia, which was deemed unacceptable. Similarly, at distances greater than 6 m, a base-out 8Δ to base-in 15Δ with diplopia, also resulting in unacceptable. Worth four-dot test (W4D) demonstrated normal far and near fusion capability. And binocular ocular motility was found to be normal (Figure 2). Both eyes exhibited clear refractive media and normal pupillary light reflex. Amblyoscope examination revealed an I + 1°, II +12°~−10°, and III (+); Assessment of near stereopsis: Fly (+), 0/3, 1/9. Fundus examination of the right eye was unremarkable (Figure 3A), whereas in the left eye, the central retinal vessels traveled straight, the macula was pulled toward the temporal side, and a macular-to-disk temporal distance of 5PD (Figure 3B). Retinoscopy revealed that the centers of the concentric circles projected onto the retina in both eyes were located in the fovea centralis, indicating that the patient fixates with the macula. Upon dilated pupil examination of the peripheral retina, the right eye exhibited no significant pathological findings (Figure 3C), whereas the left eye demonstrated fibrous membrane hyperplasia in its peripheral regions (Figure 3D). Optical coherence tomography (OCT) showed that the morphology of the left macular was fair, and there was partial separation between the layers of the neural epithelium (Figures 3E,F). Utilizing optical coherence tomography angiography (OCTA) for macular perfusion density assessment, we noted a reduced blood flow in the left macular fovea compared to the right eye, with a more pronounced decrement in nasal blood flow density (Figure 4). Fluorescein fundus angiography (FFA) revealed the right eye initially demonstrated tortuous temporal peripheral retinal vessels, whereas in the left eye, mid-peripheral retinal vessels were straight, accompanied by tortuous peripheral retinal vessels, irregular vessel diameters, and dilated capillary networks. With the progression of angiography, dilated capillaries and some vascular walls showed fluorescence leakage, culminating in late fluorescence staining (Figure 5). Based on these findings, a diagnosis was formulated: 1. MH with pseudo-exotropia of the left eye, 2. Familial exudative vitreoretinopathy, and 3. Binocular refractive error. Considering that FEVR runs in families, we performed a wide-angle fundus photography examination of the parents which did not show any significant abnormality (Figure 6). To further elucidate the diagnosis and potential genetic underpinnings, we recommended additional diagnostic modalities, including FFA and genetic testing for both the child and their parents; however, these suggestions were rejected by the patient’s parents.

## Discussion

FEVR is a genetic disease involving abnormal development of retinal vessels. Early patients with FEVR show a unique ocular morphology similar to ocular development retardation, and myopia is very prevalent in patients with FEVR and is significantly correlated with increased corneal curvature (18). Most patient with FEVR retain good visual acuity with 71% of affected patients having visual of 20/40 or better (19). Temporal dragging of the vascular arcades and heterotopia of the macula are characteristic for FEVR (8). Thus exotropia may be the first symptom in the early stages of the disease. Another feature of FEVR is disease asymmetry (20). This asymmetry can be marked with some patients having an retinal detachment in one eye and no observable signs of disease in the other.

The characteristic of this case is that the patient was first diagnosed with “exotropia.” During the consultation process, the left eye exotropia of 15° is persistent, but the alternate masking test revealed that both eyes were gazing at the target with no significant movement of eye position. Consider that strabismus is defined as one eye focusing on a target while the other eye is off-target, hence the diagnosis of pseudo-exotropia. Paracentric fixation was then ruled out by retinoscopy. Finally, fundus photography of this case found the patient had a light vitreous retinopathy, traction retinal vessel deformation, macular traction displacement, and presented with external strabismus. Electrophysiological examination showed decreased VEP amplitude and prolonged VEP latency in the left eye. Due to the large MH and displacement, the line of sight was shifted, resulting in a large positive angle Kappa similar to external strabismus. Clinically, the measurement of strabismus in patients with strabismus is preferred by prism neutralization test. However, when patients are too young to cooperate, corneal fluorescence is usually used to check the strabismus, but this method is affected by angle Kappa, which may lead to misestimation of strabismus (21).

Maintaining normal binocular monovision requires binocular normal vision and closeness, overlapping fields of view, normal retinal correspondence, straight eye positions, normal ocular motility, and normal brain fusion function. Loss of fusion function can result in true binocular axial separation, manifesting as one eye gazing at the other eye with external deviation (22). In this case, the parents requested surgical correction of the strabismus, but examination revealed that the patient had pseudoptosis and binocular retinal imaging formation corresponded, with stereoscopic vision, and surgical correction of the strabismic appearance would have resulted in the development of diplopia that was difficult to disappear (23). Therefore, the problem of strabismus appearance in this child is untreatable. The patient’s refractive error can be corrected with glasses. The fundus condition, on the other hand, should be closely followed up and treated with laser therapy if progression occurs.

Conventional FFA, the gold standard for diagnosing FEVR, provides important details about the superficial vascular system of the retina, but its visualization of the deep capillary vessels of the retina is poor (24). In contrast, the main advantage of Optical coherence tomography angiography (OCTA) is its ability to dissect the retinal vascular layer and to perform non-invasive depth-resolved imaging of retinal vessels (25). Careful examination of the contralateral eye in potentially adolescent patients is necessary to detect peripheral vascular abnormalities, and we should be more alert to the possibility of FEVR and the need to examine the peripapillary retinal vasculature (26). FEVR tends to exhibit smaller retinal artery angles, higher incidence of macular fovea dysplasia, lower retinal vessel density and vessel traction, poorer BCVA, and obvious macular abnormalities (27–30). In this case, we used OCTA to evaluate the macular blood flow density of both eyes of the patient and found that the macular fovea blood flow of the left eye was lower than that of the right eye, and the nasal side was more obvious. This is consistent with the significantly reduced macular perfusion density in the squint eye of patients with persistent external strabismus reported by Jing Zhai et al. (31). There are no previous studies on macular perfusion in patients with FEVR, but the murine models of FEVR suggests that FEVR may be a disorder of angiogenesis (3).

Therefore, in patients with FEVR, MH caused by mechanical traction of the vitreous may lead to pseudo-strabismus. In the screening of children’s visual health, the clinical features of pseudo-strabismus may be overlooked or ignored by parents or clinicians may adopt incorrect surgical treatment options for strabismus. FEVR has genetic heterogeneity, manifesting as dominant, recessive, and x-linked genetic patterns (32–34). However, in this case, the patient did not demonstrate the presence of a significant family history. Therefore, we still believe that it is important to routinely examine the fundus in outpatient clinics for patients with visual abnormalities as well as strabismus and amblyopia. In addition, molecular detection diagnostic techniques for gene mutations can provide deeper understanding of the disease and better molecular diagnosis in the special manifestations of FEVR (35). In this case report, the patient’s best-corrected visual acuity in the left eye did not reach the normal level, which may have been due to abnormalities in the microstructure of the macular of the FEVR that affected visual development (36).

## Conclusion

We believe that when a patient with pseudo-strabismus is encountered in the clinic a dilated examination of the peripheral fundus is necessary to make an early definitive diagnosis. FEVR can occur in patients without a family history, and the MH caused by FEVR may lead to pseudo-strabismus. In addition, the appearance of strabismus caused by MH is often un-operable, as surgery can lead to difficult-to-disappear diplopia in older children or adults, as well as true strabismus in infants and young children. Therefore, it is crucial to avoid incorrect treatment protocols for identifying FEVR combined with MH in the clinic.

## Data Availability

The datasets presented in this study can be found in online repositories. The names of the repository/repositories and accession number(s) can be found in the article/supplementary material.
